# Assessment of willingness to pay for expanded carrier screening among women and couples undergoing preconception carrier screening

**DOI:** 10.1371/journal.pone.0200139

**Published:** 2018-07-18

**Authors:** Elizabeth V. Clarke, Jennifer L. Schneider, Frances Lynch, Tia L. Kauffman, Michael C. Leo, Ana G. Rosales, John F. Dickerson, Elizabeth Shuster, Benjamin S. Wilfond, Katrina A. B. Goddard

**Affiliations:** 1 Center for Health Research, Kaiser Permanente Northwest, Portland, Oregon, United States of America; 2 Seattle Children’s Hospital and Research Institute, Trueman Katz Center for Pediatric Bioethics, Seattle, Washington, United States of America; TNO, NETHERLANDS

## Abstract

**Background:**

Expanded carrier screening can provide risk information for numerous conditions. Understanding how individuals undergoing preconception expanded carrier screening value this information is important. The NextGen study evaluated the use of genome sequencing for expanded carrier screening and reporting secondary findings, and we measured participants’ willingness to pay for this approach to understand how it is valued by women and couples planning a pregnancy.

**Methods:**

We assessed 277 participants’ willingness to pay for genome sequencing reporting carrier results for 728 gene/condition pairs and results for 121 secondary findings. We explored the association between attitudes and demographic factors and willingness to pay for expanded carrier screening using genome sequencing and conducted interviews with 58 of these participants to probe the reasoning behind their preferences.

**Results:**

Most participants were willing to pay for expanded carrier screening using genome sequencing. Willingness to pay was associated with income level and religiosity, but not risk status for a condition in the carrier panel. Participants willing to pay nothing or a small amount cited issues around financial resources, whereas those willing to pay higher amounts were motivated by “peace of mind” from carrier results.

**Conclusion:**

Women and couples planning a pregnancy value genome sequencing. The potentially high out-of-pocket cost of this service could result in healthcare disparities, since maximum amounts that participants were willing to pay were higher than a typical copay and related to income.

## Introduction

Genome sequencing (GS) is rapidly decreasing in cost,[1] which has led to increased investment in future medical applications of this technology, including the possibility of preconception carrier screening. In fact, expanded carrier screening is now commercially available for 100–300 conditions.[2–4] Carrier screening can provide individuals with useful information and counseling for family planning even if it does not result in reduced disease incidence.[5, 6] Because expanded carrier screening may provide information about risk to a future offspring,[7–9] it is valuable to ascertain perspectives of women and couples planning a pregnancy about personal utility of the genomic information.

Given that this approach to expanded carrier screening in the healthcare setting is fairly new in most settings, we know relatively little about how individuals perceive its use. As a result, there is a lack of priority for expanded carrier screening in mainstream healthcare practice.[5] Willingness to pay (WTP) is a contingent valuation method that is commonly used to assess how patients value new healthcare technologies. WTP assesses the maximum amount of money an individual would pay for the health intervention and still consider himself or herself better off [10] and is a commonly used method to assess personal utility of new healthcare technology to patients.

In the carrier screening setting, only a few studies have investigated pregnant women and/or couples’ WTP for this screening and have largely focused on cystic fibrosis screening, with the general consensus that patients were strongly willing to pay for this screening.[11, 12] One recent study showed that 58% of surveyed women and men of reproductive age (who were not necessarily planning a pregnancy) would be willing to pay for expanded carrier screening (via next-generation sequencing) of 50 diseases, with a median cost of €75/$89 (range: €5/$6 to €5,000/$5,921),[13] which suggests the participants value expanded carrier screening. However, it is relevant to understand the perspective of women and couples who are actually planning a pregnancy on the personal utility of this testing.[14]

To fill this important gap in the literature, we conducted the NextGen study as part of the Clinical Sequencing Exploratory Research (CSER) consortium. This study evaluated carrier status as the primary indication for GS in women and couples planning a pregnancy.[15, 16] These participants are unique in that they have a specific interest in preconception carrier testing, and they are knowledgeable about the evaluated scenarios, given their participation in the study. We hypothesized that women and couples undergoing expanded carrier screening as part of their pregnancy planning would be willing to pay for expanded carrier screening using GS because of its potential utility in terms of the long-term health of mother, partner, and offspring. We assessed specific covariates and participants’ reasoning surrounding their WTP to illuminate the rationale for their WTP (or lack thereof).

## Methods

### Study overview

The detailed study design has been published elsewhere.[16] Briefly, we identified women at Kaiser Permanente Northwest (KPNW) in the Portland, Oregon metropolitan area who were planning a pregnancy and had clinical carrier screening completed either preconception or during a prior pregnancy and were at least 6 months postpartum. If a woman joined the study, she was randomly assigned into one of two arms: genome sequencing (GS) or usual care (UC). Women randomized to the GS arm had the option to receive results for up to 728 autosomal recessive, X-linked or mitochondrial conditions and 121 medically actionable secondary findings. Women in the usual care arm were not offered testing in addition to their clinical carrier screening test.[17] Male partners were offered GS if the female partner was a carrier for at least one autosomal recessive condition.

The Kaiser Permanente Northwest Institutional Review Board reviewed and approved the study procedures. All participants provided informed consent and received written information on study procedures. This research was conducted as part of the National Human Genome Research Institute (NHGRI) CSER consortium.

### Surveys

All study participants completed an enrollment survey and a follow-up survey either 6 months after the initial enrollment visit (UC arm) or 6 months after the disclosure of carrier results (GS arm). As part of the follow-up survey, we asked participants to consider two different hypothetical situations.

“In each scenario, imagine that you are considering getting pregnant and you are discussing genetic testing with your health care provider. In both hypothetical scenarios, you are offered a choice between standard testing and genome sequencing. Standard testing will tell you about your carrier status for a small number of genetic conditions that might affect your future children. In these scenarios, your health care provider recommends that you get at least this level of genetic testing.

In the first situation, genome sequencing would provide information on carrier status for the same genetic conditions as the standard testing, and would provide additional information on other conditions that might affect your future children.

In the second situation, genome sequencing would provide the information in expanded carrier screening using GS above, but also incidental findings (also known as “secondary findings”) about genetic conditions that might affect your own future health (e.g., genetic susceptibility to cancer).”

We presented each of these hypothetical situations in sequence. Participants first completed the survey questions for the expanded carrier screening using GS alone, followed by the second scenario for expanded carrier screening plus secondary findings using GS. Participants then completed the same survey questions. For both situations, we asked participants whether they would choose standard testing or GS if provided at no cost. Using contingent valuation method, we then asked participants how much they would be willing to pay (via discrete choices) out-of-pocket for GS if they had to pay part of the cost.

### Measures

We used standardized measures for anxiety (six-item short form of the State-Trait Anxiety Inventory, STAI-6),[18] depression (PHQ-8, a version of the PHQ-9 without the suicidality item and a reliable and valid measure of depression severity),[19–23] and religiosity/spirituality (Religious Intensity subscale of the Brief Multidimensional Measure of Religiousness/Spirituality; higher score indicates less religiosity).[24] We developed a measure for a genetic knowledge index using a combination of existing measures, one from another CSER project, and edited the questions for our clinical context (S1 Table). We also modified established scales [25] to create a measure of support of science and technology (SST) (S1 Table). We modified an existing health-related quality of life (QOL) measure [26] that included the mean of QOL two items (“How would you rate your quality of life?” and “How satisfied are you with your health?”) that correlated highly (r = .61) and are both measured on 5-point scales.

The outcome of WTP was measured using seven categories of dollar amount ranges: “I would not have the test if I had to pay for it” ($0), $1–20, $21–100, $101–300, $301–600, $601–1000 and > $1000. A typical co-pay for this service at KPNW is at the lower end of this range, whereas paying for the service out-of-pocket is at the higher end of this range.

We defined couples as “at risk” if they had at least a 25% risk of having a child who could be affected by a genetic condition. Thus, “at risk” includes: (1) both partners in the couple are carriers of the same autosomal recessive (AR) condition, (2) the female partner is a carrier of an X-linked condition, and (3) either the male or the female has an autosomal dominant (AD) secondary finding. We categorized all other participants as having a “negative result with <25% risk.”

### Analysis

We limited our statistical analysis to female participants (n = 239) who reported they would choose expanded carrier screening using GS over standard testing if both were provided at no cost, because we wanted to understand WTP among those for whom the service might be applicable. We excluded participants who would not choose GS even if it did not incur an out-of-pocket expense (n = 32). Male participants were not included in the analysis because they are partners of the female respondents and are therefore not an independent population, and because there were too few follow-up survey responses to conduct a robust statistical analysis (N = 38). We did include male partners in the descriptive analysis.

We collapsed the four lowest-income categories (< $20,000, $20,000–29,999, $30,000–39,999 and $40,000–59,999) and the top three WTP categories ($301–600, $601–1000 and > $1000) for the descriptive analysis because of the small cell sizes in these categories.

We examined the associations between anxiety, depression, Genetic Knowledge Index, support of science and technology, religiosity/spirituality, education, income, race, ethnicity, having a child, quality of life, fertility patient status, risk status and WTP using multivariable ordinary least squares (OLS) regression. We modeled associations using OLS and report the unstandardized coefficients and associated 95% confidence intervals for consistency with previous studies of WTP. We tested and met the assumptions for OLS regression [27–29] and given that the outcome is technically an ordinally scaled variable, we performed a sensitivity analysis using multivariable ordinal logistic regression.

### Interviews

We conducted interviews with 58 participants in the GS arm in the last year of the study, which ranged from 12 to 18 months following receipt of their results. We approached participants with a range of results (e.g. negative result with <25% risk and at risk results), and invited both couples and females with non-participating partners. Male partners were interviewed because they also agreed to receive testing themselves. According to participant preference, interviews were conducted in-person or by phone, and couples were interviewed primarily separately but sometimes together if preferred. The interview guide included four specific WTP questions with follow up probes to elicit more detailed explanation for the given response. The first three questions asked participants whether they were willing to pay $500, $250, or $50 for expanded carrier screening using GS (including secondary findings) and their reasons why or why not. These amounts approximate a typical co-pay at KPNW at the lowest level, and an out-of-pocket cost at the highest level. The interviewer asked about each amount separately before moving to the next amount. The fourth question asked participants “Is there a maximum amount you would be willing to pay to receive these tests? Please explain.”

We recorded all interviews and transcribed them verbatim. Interview transcripts were loaded into a qualitative analysis software tool—NVIVO—and we generated code reports for the specific sub-set of WTP questions. An experienced qualitative researcher (JS) reviewed the reports multiple times to categorize participant responses and summarize the common reasons for endorsement of a given financial amount. We also conducted content analysis [30, 31] exploring the differences or similarities in WTP endorsement and related reasons by sex and at risk versus negative result with <25% risk result. Analysis summaries were continuously shared and discussed with the research team until consensus of interpretation was reached.[32]

## Results

### Survey on willingness to pay

Participants completed 309 follow-up surveys representing 269 females (114 (42.4%) GS arm, 155 (57.6%) UC arm) and 40 male partners. By design, surveys were completed about 6 months following receipt of GS results. Similar to the overall NextGen study population, the participants were mostly white, non-Hispanic, highly educated, and had high household income (Table 1).[33] Thirty-one couples (54%) were married (at follow-up) and 40% had children (at baseline).

**Table 1:** Characteristics of participants of preconception genomic carrier screening.

*Characteristic*	*Female (N = 239)*^a^	*Male Partner (N = 38)*^a^
**Mean age, yrs ±SD**	33 ±4	36 ±6
**Race/Ethnicity N (%)**		
Non-Hispanic white	189 (79)	34 (89)
**Children—yes N (%)**	96 (40)	14 (37)
**Couples married**	N = 57 couples	N = 38 couples
% Yes (of couples)	31 (54)	31 (82)
**Education N (%)**		
Less than Bachelor’s degree	50 (21)	13 (34)
Bachelor’s degree	89 (37)	18 (47)
Graduate degree	97 (41)	7 (18)
**Employment N (%)**		
Employed	208 (89)	36 (94)
Unemployed	6 (3)	1 (3)
Other (homemaker, retired, etc)	20 (8)	1 (3)
**Annual income N (%)**		
< $60,000	41 (17)	3 (8)
$60, 000 to $79, 999	38 (16)	6 (16)
$80,000 to 99,999	44 (18)	11 (29)
$100, 000 to $149, 999	72 (30)	7 (18)
≥ $150,000	34 (14)	8 (21)
**Study arm N (%)**		
Usual care	140 (59)	0 (0)
Genome sequencing	99 (41)	38 (100)
**Characteristic N (%)**^b^		
Carrier	73 (74)	26 (68)
Secondary findings	2 (2)	4 (11)

^a^Columns don’t always add up to total N because of missing data, but percentages are reflective of the total N (including missing data).

^b^Data include GS participants only (n = 99).

A total of 89% (239/269) of women and 95% (38/40) of male partners responded to the WTP survey question and indicated they would choose GS over standard testing if the GS were provided at no cost. We found no difference in WTP between the GS (94% willing to pay some amount) and usual care arms (97% willing to pay some amount). There was also no difference in WTP based on risk category (at risk, 90% willing to pay some amount versus negative result with <25% risk, 94% willing to pay some amount).

**Fig 1:**
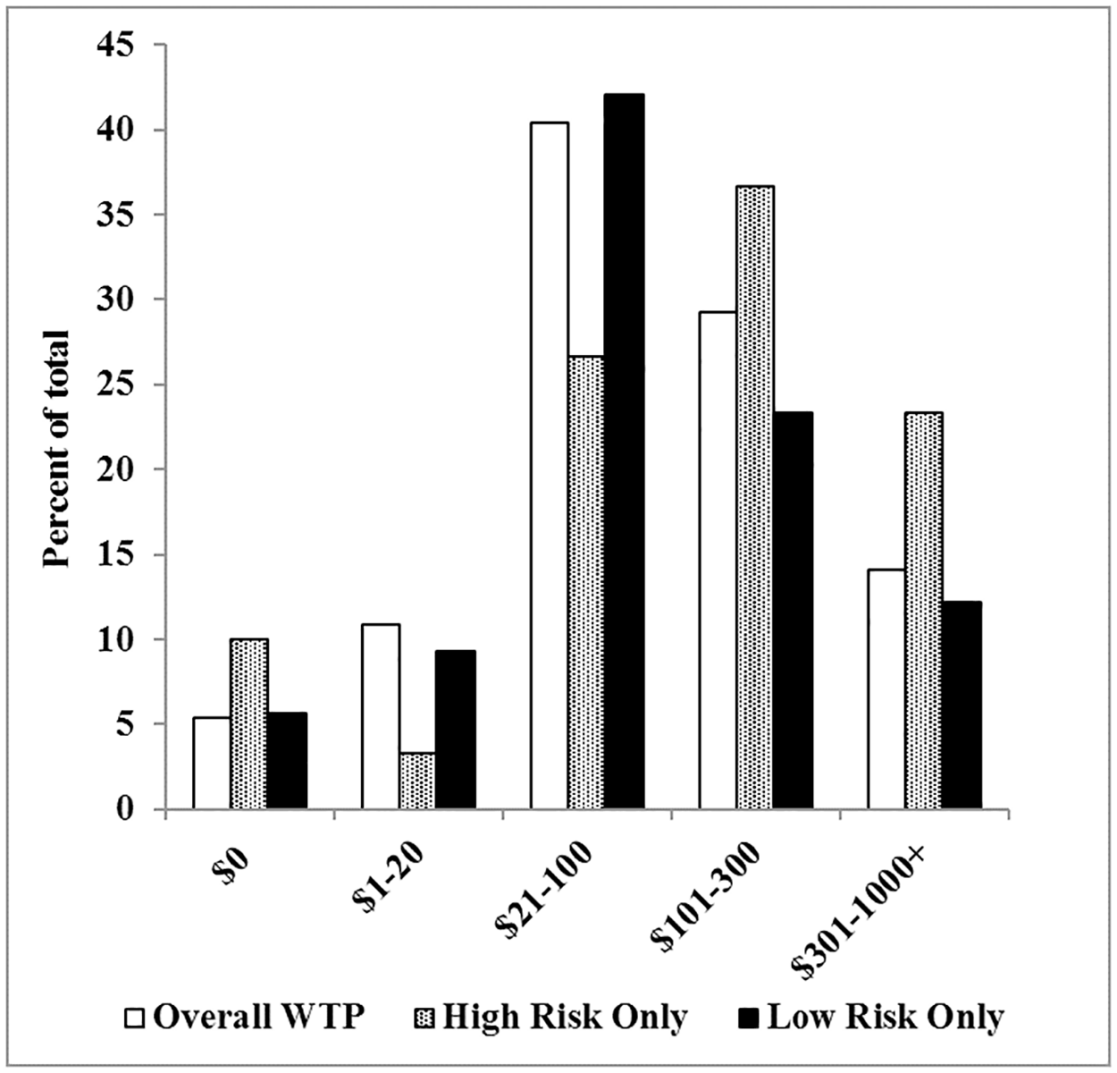
Willingness to pay for expanded carrier screening using GS (females and male partners). Survey responses to WTP questions (n = 277). Data are expressed as percent of all respondents and of the total number of at risk or negative result with <25% risk participants.

When asked “How much would you be willing to pay out-of-pocket for genome sequencing?” participant responses were very similar for the two scenarios presented in the survey. When participants were asked if they would choose expanded carrier screening using GS over standard testing if it was provided at no cost, 90% would choose GS. When asked if they would prefer expanded carrier screening plus secondary findings using GS over standard testing, 85% of participants responded they would choose GS. These results did not differ by sex (expanded carrier screening using GS: male partners: 95%; females: 89% and expanded carrier screening plus secondary findings using GS: male partners: 95%; females: 83%) or study arm (expanded carrier screening using GS: GS arm: 94%; UC arm: 97% and expanded carrier screening plus secondary findings using GS: GS arm: 95%; UC arm: 96%). The data reported for the rest of this study all refer to expanded carrier screening (without secondary findings) using GS since there were not meaningful differences between the responses to the two scenarios. Overall, participants were overwhelmingly willing to pay some amount for GS versus not willing to pay (Fig 1), and only 4% of women and 13% of male partners were not willing to pay for GS.

Female and male partner participants’ WTP was highest at the $21–100 and $101–300 levels, respectively (Fig 1).

We found the distribution of the residuals to be approximately normally distributed and homoscedastic after performing ordinary least squares (OLS) regression for women only and found that an ordinal logistic regression lead to the same inferences. Thus, for more ease of interpretation, we report the results of the OLS model. The amount participants were willing to pay was related to income (p < .01). Individuals in all categories below $150,000 annual household income were willing to pay less than those earning $150,000 or more (Table 2). People who were more religious/spiritual were willing to pay less than those who were less religious/spiritual (p = .01). No other covariates were significantly associated with WTP (Table 2).

**Table 2:** Multivariable linear regression results of willingness to pay for expanded carrier screening for GS in women (n = 239).

*Variable*	*b*^a^	*95% confidence interval*		*p*
		LB	UB	
Anxiety	<0.01	-0.01	0.02	.84
Depression	0.02	-0.03	0.07	.48
General knowledge index	-0.63	-1.52	0.26	.16
Support of science and technology	0.12	-0.16	0.40	.40
Religious intensity	0.22	0.05	0.39	.01
Education				
Less than bachelors degree	Reference category			
Bachelors degree	0.19	-0.22	0.61	.36
Graduate degree	-0.07	-0.51	0.37	.76
Income				
$0 to $59, 999	-1.14	-1.68	-0.60	< .01
$60, 000 to $79, 999	-0.75	-1.27	-0.23	< .01
$80, 000 to $99, 999	-0.91	-1.40	-0.42	< .01
$100, 000 to $149, 999	-0.93	-1.38	-0.48	< .01
<$150,000	Reference category			
White (0 = no, 1 = yes)	0.49	-0.11	1.09	.11
Hispanic (0 = no, 1 = yes)	-0.40	-1.01	0.21	.20
Child (0 = no, 1 = yes)	-0.28	-0.60	0.03	.08
QOL	0.12	-0.12	0.35	.30
Infertility	-.08	-0.39	0.23	0.61
Risk status	2.62	-0.42	0.72	0.61

b^a^ = regression coefficient.

### Interviews about willingness to pay

We conducted 58 interviews with 36 females and 22 male partners, representing 35 individuals with negative results with <25% risk and 23 individuals with at risk results. In exploring the responses to the specific amounts of $50, $250 and $500, most participants (90%; 34 females and 18 male partners) were willing to pay $50 for testing, expressing this to be a very reasonable amount similar to co-pays for other services. The willingness to pay $50 for testing was endorsed as acceptable regardless of sex or risk status. In fact, most participants indicated they might regret not obtaining GS for $50. For more detail on WTP specific amounts of $50, $250, and $500 please see S1 Table.

The majority of participants interviewed were willing to pay between $250–350. There were no clear differences between females and male partners in their maximum WTP responses and reasons given for these amounts. Interestingly, at risk participants (but not participants with a negative result with <25% risk) were willing to pay higher amounts overall, particularly at the $500 and more than $1000 levels. Table 3 shows the maximum amounts that individual participants were willing to pay, by sex and risk status, along with reasons why.

**Table 3:** Interviewed participants’ maximum amount willing to pay for expanded carrier GS and reasons.

Maximum Amounts WTP (open ended question)							
Max Amounts	Total	Female (36)		Male (22)			
	N (58)	FNR (23)	FAR (13)	MNR (12)	MAR (10)	Reasons Why (could be more than one)	Illustrative Quotes
***$0***	**2** **(3%)** (couple)		1		1	► Would not pay unless free or had very compelling reason to do so / cost not worth value.	Honestly, we would not have done it if it cost any money.–At risk female
$100–249*	**11** **(19%)** *7 F* *4 M*	4	3	2	2	► Anything more is too expensive given current finances. ► Would need a reason to pay more.	I don’t make a lot of money…health care is something I hate to spend money on unnecessarily.–Female with a negative result with <25% risk I think $150 is the maximum I’d be willing to pay with my situation. If I had a history of my family having genetic disorders of some sort then I might be willing to pay more.–At risk female
***$250–350***	**20** **(35%)** *14 F* *6 M*	11	3	5	1	► Not too out of reach and could make “work” to alleviate any worry or anxiety.	**Wife**: Two-fifty is reasonable in my mind—… As long as I was interested in having kids I would pay. I would without hesitation I’d pay as much as $250. **Husband:** Yeah, that seems like a fair price to me. I still would only do it if I was going to have kids for sure at that price. I wouldn’t spend more just on a whim.–Couple with a negative result with <25% risk The reason to pay $250 would be to kind of alleviate some of those worries, particularly about our child’s potential health…And just generally we’re pretty solidly middle class so spending more than $250 on something that we’re not a hundred percent sure that is necessary or whatever can seem out of reach.–Male with a negative result with <25% risk
***$500***^†^	**11** **(19%)** *6 F* *5 M*	4	2	2	3	► Affordable enough amount to do it to receive benefit of test knowledge and genetic counseling. ► Would need to convince partner of this amount.	I like having the answers. For me, $500 would be worth finding out, and being able to map our plan going forward, answer any questions we might have, or if there was anything we could do to prevent anything. I feel like having those answers would give us the push forward to do that.–At risk female I would do $500 because the testing really helped me on a cognitive behavioral level [regarding fears] because it replaced all the unknown whys…but my husband would be different and say, ‘Nah, it will be okay,’ until after my pleading, he’d say, ‘okay.’–Female with a negative result with <25% risk
***$100 or more***	**14** **(24%)** *8 F* *6 M*	4	4	3	3	► A reasonable amount for the service. Less than other reproductive or family planning services. ► Learning about potentially medically actionable results for offspring or oneself is “priceless”.	“For peace of mind…[finding] medically actionable results that I could then act on…would be priceless.”–Female with a negative result with <25% risk **Wife**: It’s worth a lot more than five hundred dollars to me. **Husband:** I mean, this is talking about future health—I’d be willing to pay a lot of money. So we both agree, we both think like two grand, in a heartbeat.–Couple with a negative result with <25% risk

FNR, female negative result with <25% risk; FAR, female at risk; MNR, male negative result with <25% risk; MAR, male at risk.

* Participants were willing to pay exactly $100 to $150 as their stated maximum amount.

^**†**^ Participants were willing to pay exactly $500 as their stated maximum amount.

In general, factors that were considered included current financial situation, whether there was a compelling reason to pay more (e.g., family history or health concern), and whether they felt their partner would be willing to pay this amount. The potential benefits considered to justify paying more included alleviation of anxiety in reproductive planning, the potential benefits of test knowledge and genetic counseling, and believing that learning about potentially medically actionable results for offspring or oneself is “priceless.”

## Discussion

Among the NextGen participants who indicated they would receive expanded carrier screening using GS if no additional cost were incurred, we found that females and male partners value expanded carrier screening using GS, as measured by their WTP for this service. Those who were willing to pay higher amounts reasoned that the cost of the GS was justified by the information gained and would alleviate worry and anxiety. This suggests that, at least in the population studied, couples planning to have a child found personal utility in this service to reduce concern or worry about the health of their potential offspring and to benefit from knowing what to expect (Table 3). It is important to document these measures of personal utility, because this is a critical factor underlying one of the rationales for providing carrier screening services.

Of note, many participants in the interviews were open to paying a higher amount (±$250) when they considered other factors, such as partner willingness, a doctor’s recommendation, or lack of knowledge about their own or their partner’s family history (Table 3). Given that these and other factors/motivations surrounding one’s WTP [34–36] are not necessarily static, participants not willing to pay for medical screening at one point in time may not translate into an unwillingness to pay at all. As more information about GS becomes available and people learn about it and/or have family members who have such tests, WTP may change.

The data suggest some differences between those who were willing to pay more than others. Not surprisingly, participants at the highest income level were willing to pay more than participants at all the other income levels, and hesitancy to pay higher amounts for carrier GS was typically related to limited financial resources. This relationship between income and contingent valuation for such testing is consistent with the literature. A recent systematic review of WTP for diagnostic technologies found that among 66 relevant studies published from 1985–2011, higher income (among other variables) was generally associated with a higher WTP for diagnostic information.[37]

Importantly, amounts participants were willing to pay were substantially higher than a typical co-pay (although the amounts they were willing to pay are less than what the test may currently cost). We found that amounts were related to income and potentially the ability to pay high out-of-pocket costs, thus our findings should be considered in terms of the risk for creating health disparities related to financial resources. Therefore, the present results we present here demonstrating the value of this service could help inform clinical and insurance policy about coverage of GS for preconception screening.

We evaluated whether there was an association between at risk status based on test results, and willing to pay higher amounts in both the survey (6 months after result disclosure–Table 2) and interview data (12 to 18 months after result disclosure–Table 3). For the survey questions, this association was not significant, implying that risk status is not necessarily factored into how much participants value expanded carrier testing, as has been reported previously in the case of WTP for genetic testing for Alzheimer’s Disease.[38] As noted in the interviews, participants receiving a negative result with <25% risk may have valued the expanded carrier screening using GS as much as those who had an at risk result given that they felt the test gave them “peace of mind” and alleviated worry about the future health of their child. Further, based on the genes included under the definition of “at risk” for the study, there was variability in the risk of both developing the disease and the disease severity, which could influence participant valuation of the test, as has been previously shown in the case of individuals’ WTP for diagnostic technologies.[37] For example, our definition of “at risk” included *HFE* (hereditary hemochromatosis with one partner carrying the C282Y allele and one carrying the H63D allele), *BRCA1* (breast cancer) and *F8* (hemophilia) results, which each confer very different levels of risk for the participant or their future children. Future studies should evaluate whether different types of inheritance and risk level influence perceptions of clinical utility surrounding the long-term health of the individual, couple and future offspring.

Interestingly, participants who reported a lower extent of religiosity/spirituality were willing to pay a higher amount. This is likely because people who have a higher extent of religiosity are less willing to pay, because they do not see the benefit of screening since for these people the test results typically do not impact reproductive choices.[13, 39, 40] Among the few studies that have evaluated the relationship between religiosity and WTP for genetic risk assessments, one report found that women who considered themselves religious were less likely to pay for invasive prenatal diagnostic testing, similar to our findings.[41] The relationship between religiosity and WTP for expanded carrier screening may be particularly important in pre-conception and prenatal care given that religious beliefs may play a role in decisions regarding reproductive decisions compared with other medical decisions.

Study participants were identified because they had been offered preconception carrier screening by their usual provider and they received GS as part of study participation. Patients receiving preconception carrier screening were more likely to receive services for infertility than in a typical pregnancy, including 41% percent of study participants. Since reproductive services can include costly treatments that are often not a reimbursed covered benefit, participants receiving these services may have been willing to pay more than the average patient. However, there was no difference in WTP between patients receiving infertility services and the other participants (Table 2).

This study included responses that are provided in the context of individuals who actually received the service, in contrast to prior studies, which evaluated WTP for hypothetically receiving the service.[7, 13, 14, 34, 37, 38, 42, 43] This testing may not be relevant to everyone of reproductive age, so while our study may not reflect the perspectives of the entire adult population, it is focused on an appropriate subset.

The secondary findings returned to participants were negative for nearly everyone; thus, the added importance of these results for the small number of people for whom this information was valuable was not evident among the overall population studied. Therefore, the results from this study cannot clearly identify whether there is added value from return of secondary findings.

Our study reveals that participants found preconception expanded carrier screening using GS to have personal utility as demonstrated by their WTP for this screening. Since participants made decisions about which results to receive, the results had clear personal meaning for couples.[44, 45] While the information they received did not necessarily have direct clinical utility, participants noted that expanded carrier screening using GS was valuable to them because it alleviated concerns and/or helped them gain knowledge/understanding about their potential child’s health, similar to other studies identifying factors that contribute to personal utility derived from GS results.[46, 47] Because the findings that were returned to participants revealed information about consequential and serious medical conditions, the “peace of mind” that participants felt after receiving their results (regardless of risk status) could translate into a psychosocial benefit. Moreover, the results could help inform reproductive decision making. Therefore, it can be argued that the value participants gained from testing could also have clinical benefit in terms of relieving anxiety and promoting informed decision making.[45]

### Limitations

Our investigation is a pilot study of WTP for expanded carrier screening as part of the NextGen study. Thus, we did not have an exhaustive evaluation of WTP for this technology. It should also be noted that participants were not diverse, and consisted of largely moderate to higher-income, well-educated, white individuals who indicated they would receive expanded carrier screening using GS if no additional cost were incurred. Our study population may not be representative of everyone who would want pre-conception carrier screening, so our results must be balanced with the potential for creating healthcare disparities, since maximum WTP amounts were related to income. This is important because earlier studies evaluating WTP in a more general population of women and couples of reproductive age and/or planning a pregnancy have shown that this broader population values expanded preconception carrier screening.[13, 14] Because this study only evaluates WTP for those who would potentially use expanded carrier screening using GS, further research exploring the value, benefits and disadvantages which includes the views of those who would not be willing to pay for this service is needed to help inform the debate about expanded carrier screening using GS. In addition, our analysis was limited given that we evaluated WTP as a categorical rather than a continuous variable, participants’ WTP for expanded carrier screening using GS was only assessed after receiving their results, male partners were selected based on females’ participation in the study, and family history was not directly measured.

### Conclusions

Our study provides novel information and fills a gap in our understanding of WTP for expanded preconception carrier testing using GS. Our approach of following survey data with qualitative interviews to probe participants’ valuation of testing is advantageous because it has enabled us to provide context for understanding why participants were willing to pay certain amounts for the technology. Indeed, it can be argued that an in-person interview may capture more information than a survey,[48] so having both the interviews and survey data strengthens the reliability of our results. As GS becomes more widespread and more commonly offered in clinical practice, it is important to consider how patients value this new information. Future research about WTP in a broader population could help to provide a more comprehensive picture of how different groups of patients may value results from GS including expanded carrier screening.

## Supporting information

S1 Table Analysis of WTP for expanded carrier screening using GS among women and male partner participants at three levels ($50, $250 and $500). (PDF)

S2 Table Genetic knowledge index (A) and support of science and technology scale questions from follow-up survey (B). (PDF)
