# Supplementary material for: Assessment of willingness to pay for expanded carrier screening among women and couples undergoing preconception carrier screening
Source: PLoS One. 2018 Jul 18;13(7):e0200139. doi: 10.1371/journal.pone.0200139 (PMC6051630; doi:10.1371/journal.pone.0200139)
Supplement: S1 Table — (PDF) [file pone.0200139.s001.pdf]

S1 Table.

Analysis of WTP for expanded carrier screening using GS among women and male partner participants at three levels ($50, $250 and $500).

**WILLING TO PAY $50**						
**Themes**	**Total**	**Female (36)**		**Male (22)**		**Most Common Reasons Given** *(may endorse more than one reason)*
	**N (58)**	**Lower (23)**	**Higher (13)**	**Lower (12)**	**Higher (10)**	
Yes – Expressed definite enthusiasm	**52 (90%)** *34 F 18 M*	22	12	10	8	**► Very reasonable amount (like co-pay)/equivalent to what pay for other services like lab fees**<br>**► Very worth the money to know information / might regret not obtaining test for this amount**
Maybe – Expressed some hesitancy	**6 (10%)** *2 F 4 M*	1	1	2	2	**► Requires a "reason": doctor recommendation, family history or health concern**<br>**► Amount seems almost too low given the nature of the services/ might make one question validity of results**
No – Expressed unlikeliness	**0**	0	0	0	0	

**WILLING TO PAY $250**						
**Themes**	**Total**	**Female (36)**		**Male (22)**		**Most Common Reasons Given** *(may endorse more than one reason)*
	**N (58)**	**Lower (23)**	**Higher (13)**	**Lower (12)**	**Higher (10)**	
Yes – Expressed definite enthusiasm	**36 (62%)** *23 F 13 M*	15	8	7	6	**► Fair amount likely affordable for most**<br>**► Reasonable amount for benefit of knowledge gained and "peace of mind"**
Maybe – Expressed some hesitancy	**12 (21%)** *7 F 5M*	4	3	4	1	**► More likely if had a "reason": doctor recommendation, family history or health concern**<br>**► More likely to pay this amount with less need for deliberation with self or spouse**
No – Expressed unlikeliness	**10 (17%)** *6 F 4 M*	4	2	1	3	**► Still too much money for income level without a specific reason or need**<br>**► Too much because it would not change reproductive plans**<br>**► Prefer not to spend discretionary income on medical costs**

WILLING TO PAY $500						
**Themes**	**Total** N (58)	**Female (36)**		**Male (22)**		**Most Common Reasons Given** *(may endorse more than one reason)*
		**Lower (23)**	**Higher (13)**	**Lower (12)**	**Higher (10)**	
Yes – Expressed definite enthusiasm	**17 (28%)** *10F 7 M*	5	5	4	3	**► Knowledge gained worth cost for "peace of mind"**<br>**► This amount "pops" into mind as a reasonable amount**<br>**► Appropriate cost given older age during reproductive planning**
Maybe – Expressed some hesitancy	**9 (16%)** *6F 3M*	3	3	1	2	**► Requires a "reason": doctor recommendation, family history or health concern**<br>**► Requires more discussion with partner to consider amount**
No – Expressed unlikeliness	**32 (56%)** *20F 12M*	15	5	7	5	**► Need a more specific reason or concern: doctor recommendation, family history or health concern**<br>**► Not unreasonable amount but unaffordable given current income**<br>**► Too much because it would not change reproductive plans**<br>**► Other health care needs (e.g., medications) a higher priority**
