# Supplementary material for: Assessment of willingness to pay for expanded carrier screening among women and couples undergoing preconception carrier screening
Source: PLoS One. 2018 Jul 18;13(7):e0200139. doi: 10.1371/journal.pone.0200139 (PMC6051630; doi:10.1371/journal.pone.0200139)
Supplement: S2 Table — (PDF) [file pone.0200139.s002.pdf]

S2 Table. Genetic Knowledge Index (A) and Support of Science and Technology Scale Questions From Follow-Up Survey (B).

**A. Genetic Knowledge Index Questions From Follow-Up Survey**
(1) A genome sequencing test for carrier status can tell me if I will have a child with Down syndrome.
(2) A negative (or normal) result for carrier status from a genome sequencing test means that I will not have a child with a serious genetic condition.
(3) Genome sequencing tests may find mutations in my genes that I can pass on to my children.
(4) Genome sequencing tests can tell me about my risks of developing several different diseases.
(5) Genome sequencing tests may find mutations that determine how I respond to certain medicines.
(6) If a woman's genome sequence test shows she is carrier for an x-linked genetic condition, her sons have a risk of having that condition.
(7) If my genome sequence test shows I am a carrier for an autosomal recessive genetic condition, my partner must also be a carrier for the same condition in order for us to have a chance of having a child with the condition.
(8) If my genome sequence test shows I am carrier for a genetic condition, my relatives may also be carriers for the same genetic condition.
(9) It is unlikely that both partners in a couple are carriers for the same condition.
**B. Support of Science and Technology Scale Questions From Follow-Up Survey**
(1) It is important for me to know whether I have a mutation that predisposes me to a health condition.
(2) It is important for me to know whether a child who I conceive has a genetic based health condition.
(3) Knowing if my partner and I each carry a genetic mutation in the same gene is valuable information.
(4) Knowing my genetic carrier status results will help me make better decisions about having children in the future.
(5) Everyone in the United States should be offered genome sequencing for carrier status.
(6) I received enough information to make a decision regarding the category of results I chose to receive (e.g., serious, mild, secondary findings).
(7) People should not be screened for carrier status if they do not request it.
(8) I do not trust modern medicine.
(9) I would like my doctor to have access to the most updated technology.
(10) I would want my doctor to know the results of my genome sequencing.
